# Septic Sternoclavicular Arthritis: An Uncommon Manifestation in the Context of Immunosuppression Following SARS-CoV-2 Infection

**DOI:** 10.7759/cureus.49609

**Published:** 2023-11-28

**Authors:** Frederico Silva, Maria Margarida Rosado, Inês G Simões, Bruno L Correia

**Affiliations:** 1 Internal Medicine, Centro Hospitalar Universitário do Algarve - Unidade de Portimão, Portimão, PRT; 2 Orthopaedics, Centro Hospitalar Universitário do Algarve - Unidade de Portimão, Portimão, PRT

**Keywords:** sars-cov-2 complications, multidisciplinary approach, covid-19-induced immunosuppression, septic arthritis, sternoclavicular joint

## Abstract

This case report discusses a rare occurrence of septic arthritis in the sternoclavicular joint (SCJ) following SARS-CoV-2 infection-induced immunosuppression in a 94-year-old patient. Despite its rarity, the case underscores the importance of recognizing unusual manifestations of COVID-19, emphasizing the need for healthcare providers to consider COVID-19-induced immunosuppression in differential diagnoses. Swift diagnosis, surgical intervention, and appropriate antibiotics led to a favorable outcome, highlighting the significance of a multidisciplinary approach.

## Introduction

Septic arthritis of the sternoclavicular joint (SCJ) is a rare condition, accounting for less than 1% of all osteoarticular infections. It is often associated with various risk factors (diabetes mellitus, end-stage renal disease, intravenous drug use, trauma, rheumatoid arthritis, intra-articular injections, crystalline arthropathies, radiation therapy, cirrhosis, joint surgery, skin infections near the joint, malignancy, chemotherapy, and tracheostomy), with immunosuppression being the most prevalent. Delayed treatment can lead to severe complications, such as osteomyelitis, mediastinitis, and empyema [1]. COVID-19 has been associated with immune system dysregulation, potentially leading to immunosuppression in affected individuals [2].

There have been documented cases of reactive arthritis and the development of inflammatory conditions, such as rheumatoid arthritis, psoriatic arthritis, and gouty arthritis following COVID-19. However, instances of septic arthritis occurring during or after COVID-19 are relatively scarce [3].

Understanding the implications of COVID-19-induced immunosuppression is paramount in medical practice. Being aware of this phenomenon and integrating it into differential diagnoses is pivotal for accurate patient assessments and optimal healthcare outcomes.

## Case presentation

A 94-year-old female presented to the emergency department with the acute onset of worsening fever and prostration. Upon review of symptoms, she was found to have chills and fever for about a week, along with swelling in the anterior chest region for the past five days. Her past medical history included chronic lower back pain, high blood pressure, dyslipidemia, and osteoporosis. In a more recent occurrence, the patient was hospitalized for nine days due to a severe SARS-CoV-2 infection and was discharged two weeks prior to this current emergency situation. The patient had completed the full vaccination regimen and received two extra booster doses. The latest booster was administered three months before admission. The severity of the COVID-19 infection was deemed severe due to observed pneumonia and peripheral oxygen saturation below 90%. During the recent hospital stay, the treatment for COVID-19 included a two-day course of dexamethasone 6 mg per day along with supplemental oxygen.

**Figure 1:**
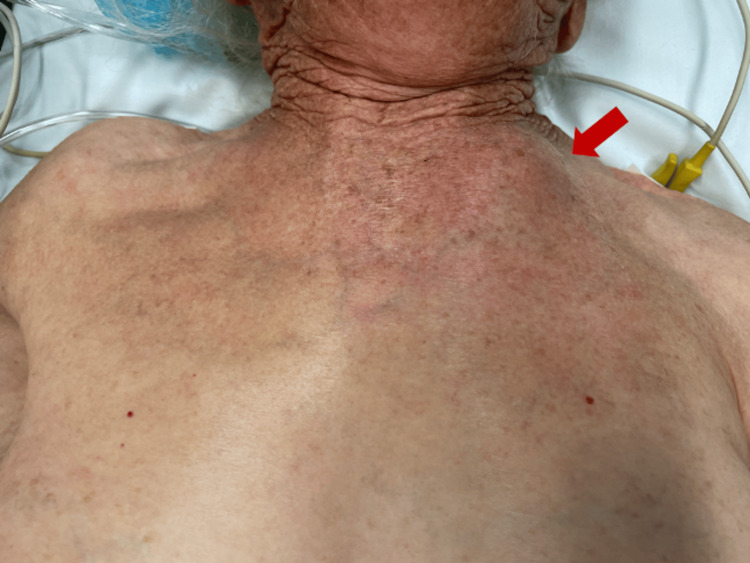
Appearance of the left sternoclavicular joint prior to surgical intervention.

On physical examination, the patient exhibited fever and a firm, elastic mass in the left SCJ area, which was warm to the touch, red, and tender upon palpation (Figure 1).

Initially, there was concern for sepsis with severe complication due to progression of likely septic arthritis. The patient denied any history of shortness of breath, nausea, vomiting, abdominal pain, or hematic losses at admission. Laboratory analysis of the blood showed leukocytosis 20.2 x 10E3/uL (4.0-10.0 x 10E3/uL) with neutrophilia 16.9 x 10E3/uL (2.0-7.0 x 10E3/uL) and elevated C-reactive protein levels 305.3 mg/L (0.0-5.0 mg/L) supporting the hypothesis of an infectious disease causing the preliminary findings described.

**Figure 2:**
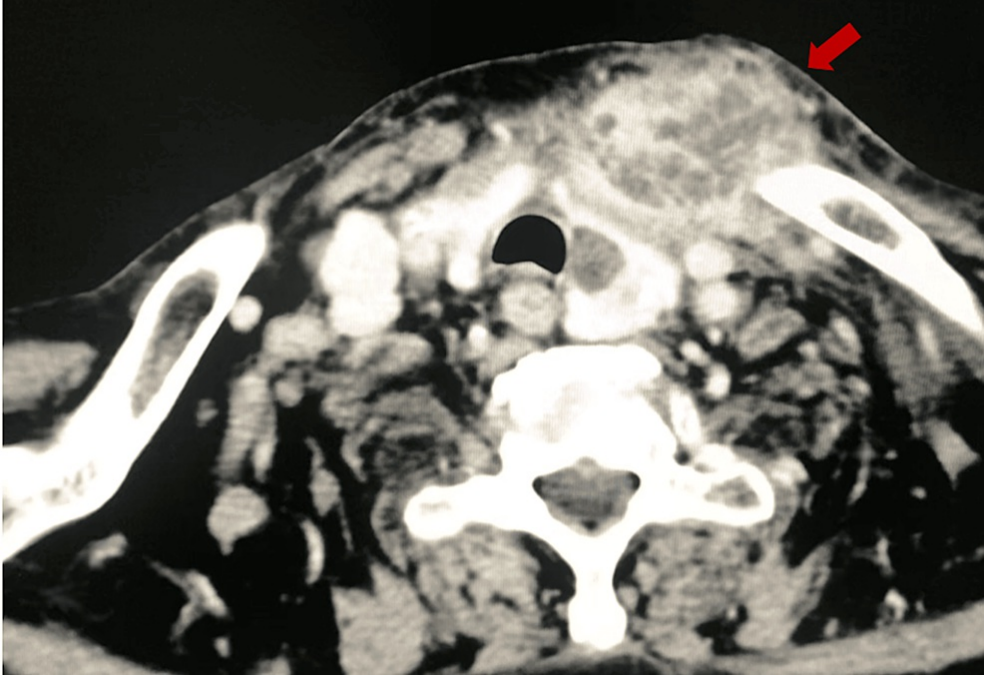
Computed tomography (axial view) reveals a multiloculated abscess impacting the right sternoclavicular joint.

Soft tissue ultrasound revealed a joint effusion in the left SCJ, with edema and heterogeneous adjacent tissues. A hypoechoic image suggestive of a periarticular collection was also observed. Computed tomography (not contrast-enhanced) confirmed the presence of a multiloculated abscess with intra-articular continuity measuring approximately 5.3 x 5.2 x 3.8 cm (Figure 2).

The patient was admitted with a diagnosis of septic arthritis of the SCJ and initiated empirical antibiotic therapy with vancomycin 1200 mg intravenously every eight hours, equivalent to 15-20 mg/kg. On the second day, ultrasound-guided drainage was attempted, resulting in the evacuation of purulent content. However, due to the presence of an organized collection, this approach proved ineffective. Consequently, on the third day, open surgical drainage was performed through arthrotomy of the SCJ, followed by irrigation and surgical debridement. Bacterial examination of the collected material identified, on the sixth day since admission, methicillin-sensitive *Staphylococcus aureus* (MSSA), prompting a switch in antibiotic therapy to flucloxacillin (2 g intravenously every six hours). No bacteremia was identified on blood cultures, and no evidence of infective endocarditis was found on transthoracic echocardiogram. Altogether, the patient underwent an eight-week antibiotic regimen, with the initial three weeks administered intravenously, followed by the subsequent five weeks taken orally. Following an uncomplicated hospital stay post-surgery, the patient was released with a prescription for oral antibiotics to finalize the recommended extended antibiotic therapy [4]. Later outpatient visits (appointments scheduled every two weeks for a span of four months) showed symptom resolution, complete recovery from the condition, and laboratory improvement with a decrease in inflammatory markers.

## Discussion

This case report aims to highlight the development of septic arthritis of the SCJ, a joint rarely affected by this condition, in a patient who experienced SARS-COV-2 infection and subsequent immunosuppression. The interplay between the immunosuppressive effects of COVID-19 and the patient's medical history likely contributed to the favorable environment for the development of septic arthritis.

The literature increasingly highlights the connection between COVID-19 and septic arthritis, although it remains relatively uncommon. Further research is crucial to fully comprehend this association. Importantly, immune system suppression, whether induced by immunosuppressant drugs or autoimmune conditions, stands out as a well-established key risk factor for septic arthritis development [5].

Immunocompromised states significantly increase the risk of SCJ infections, alongside factors, such as diabetes mellitus, end-stage renal disease, and intravenous drug use. Trauma, central venous catheters, rheumatoid arthritis, and intra-articular injections are common risk factors. In addition, suprapubic catheters, crystalline arthropathies, radiation, cirrhosis, joint surgery, skin infections, malignancy, chemotherapy, breast cancer and radiation, tracheostomy, epidural block, and coronary angiography contribute to this risk. Intriguingly, 23% of cases present without any identifiable predisposing factors [1].

Extensive research has explored COVID-19's impact on host immune responses, revealing its suppression of both innate and adaptive immunity. One potential pathway is the direct harm the SARS-CoV-2 virus inflicts on joints, possibly by infecting and damaging cells in the synovial membrane lining these joints. Another theory suggests that COVID-19 weakens the immune system, making it harder for the body to fight off infections. This occurs due to the virus inducing inflammation and damaging white blood cells, vital for infection defense. In addition, COVID-19 can trigger secondary complications, such as pneumonia, leading to bacteremia, which provides a pathway for bacteria to enter joints. Treatment strategies, including corticosteroids and tocilizumab, used in managing COVID-19, can further contribute to the immunosuppressed state, increasing the risk of septic arthritis [6,7].

In our patient, SCJ septic arthritis was a diagnosis that was quickly established, due to the history of fever, the compatible findings in physical examination, and the presence of the most common risk factor: immunosuppression.

Despite the rarity of this condition, a comprehensive physical examination, detailed medical history, and a multidisciplinary approach played key roles in achieving a favorable outcome.

## Conclusions

In essence, this case report highlights the pressing importance of identifying uncommon manifestations and lasting complications of COVID-19, specifically septic arthritis, in patients with weakened immune system. It underscores the intricate nature of the virus and its varied impact on various organ systems, even beyond the acute phase of the disease. Healthcare providers need to maintain a high level of suspicion and consider COVID-19 as a potential culprit when confronted with unusual symptoms in immunocompromised individuals. Swift diagnosis and appropriate management are paramount, necessitating a multidisciplinary approach and ongoing research to enhance our understanding of the diverse ways in which COVID-19 can manifest. By staying vigilant, raising awareness, and fostering collaborative efforts among healthcare professionals, we can enhance outcomes for immunocompromised patients and the broader community.
